# Medicinal Formula Huazhi-Rougan Attenuates Non-Alcoholic Steatohepatitis Through Enhancing Fecal Bile Acid Excretion in Mice

**DOI:** 10.3389/fphar.2022.833414

**Published:** 2022-06-01

**Authors:** Chunlin Li, Siyu Yu, Xiaoxiao Li, Ying Cao, Meng Li, Guang Ji, Li Zhang

**Affiliations:** Institute of Digestive Diseases, Longhua Hospital, Shanghai University of Traditional Chinese Medicine, Shanghai, China

**Keywords:** huazhi-rougan formula, non-alcoholic steatohepatitis, bile acid excretion, gut microbiota, ileal bile acid transporter

## Abstract

Huazhi-Rougan (HZRG) formula is a Traditional Chinese medicine prescription, and has been widely used to treat non-alcoholic fatty liver disease (NAFLD) and its progressive form non-alcoholic steatohepatitis (NASH). However, the anti-NASH effects and the underlying mechanisms of HZRG have not yet been characterized. Here we showed that 4-week HZRG treatment alleviated methionine-choline-deficiency (MCD) diet-induced NASH in C57BL/6J mice, as evidenced by the improvement of hepatic steatosis and inflammation, as well as the decrease of serum levels of alanine and aspartate transaminases. Fecal 16S rDNA sequencing indicated that HZRG reduced the enrichment of pathogenic bacteria and increased the abundance of bacteria gena that are involved in bile acid (BA) conversation. The alteration of fecal and serum BA profile suggested that HZRG enhanced fecal BA excretion, and reduced the reabsorption of toxic secondary BA species (LCA, DCA, HCA). We further analyzed the BA receptors and transporters, and found that HZRG inhibited the expression of ileal bile acid transporter, and organic solute transporter subunit β, and increased the expression of intestinal tight junction proteins (ZO-1, Occludin, Claudin-2). The modulation of gut dysbiosis and BA profile, as well as the improvement of the intestinal environment, may contribute to the decrease of the p-65 subunit of NF-κB phosphorylation, liver F4/80 positive macrophages, inflammatory cytokine IL-1β and TNF-α expression. In conclusion, HZRG treatment enhances fecal BA excretion *via* inhibiting BA transporters, modulates BA profiles, gut dysbiosis as well as the intestinal environment, thus contributing to the beneficial effect of HZRG on NASH mice.

## Introduction

Nonalcoholic fatty liver disease (NAFLD) is emerging as the leading chronic liver disease, and is considered the hepatic manifestation of metabolic syndrome, which affects more than a quarter of the world population (Younossi et al., 2018). Nonalcoholic steatohepatitis (NASH) is the progressive form of NAFLD, and is characterized by liver steatosis, inflammation, with or without fibrosis. NASH plays a pivotal role in the progression of metabolic syndrome and the development of certain tumors, thus attracting numerous pharmaceutical companies to be active in the drug development market (Anstee et al., 2019). There are many drugs in the pipeline that hold promise for treating NASH, however, approved pharmacological therapy for NASH is not available due to the complicated pathophysiological mechanisms (Negi et al., 2022). Therefore, new treatment strategies for NASH are urgently needed.

Bile acid (BA) receptors emerged as promising drug targets for NASH in recent years (Friedman et al., 2018). BAs are solely synthesized in the liver, and are considered to be associated with the pathogenesis and management of NASH. BAs are detergent molecules that aid in fat and vitamin absorption. BA synthesis takes place in the liver from cholesterol, and occurs *via* both classical and alternative pathways. The classical pathway is initiated by the enzyme cholesterol 7α-hydroxylase (CYP7A1), and physiologically accounts for approximately 75% of BA production, whereas the alternative pathway is catalyzed by the enzyme sterol-27-hydroxylase (CYP27A1), and contributes about 25% of BA production. Chenodeoxycholic acid (CDCA) and cholic acid (CA) are primary BAs produced in humans, and their ratio is determined by the enzyme sterol 12α-hydroxylase (CYP8B1). CDCA in mice can further generate muricholic acids (MCAs) (Gustafsson et al., 1981). Primary BAs are then conjugated with glycine or taurine in the hepatocytes, stored in the gall bladder, and released into the duodenum upon fat ingestion. BAs facilitate fat absorption within the ileum. After conducting their functions, most BAs are reabsorbed in the distal ileum *via* the ileal bile acid transporter (IBAT), while the remains are excreted into the colon where billions of bacteria and microorganisms are colonized. The microbial metabolism of BAs begins in deconjugation, removing the taurine or glycine from BAs, this process is conducted by bile salt hydrolase-producing bacteria. The deconjugation of BAs is of great importance because it can counteract BA toxicity and promote secondary BA production in the colon. In humans, lithocholic acid (LCA) that derived from CDCA and deoxycholic acid (DCA) from CA are the two major secondary BAs, while MCA can be further converted into hyocholicacid (HCA) and hyodeoxycholic acid (HDCA) in rodents (Wahlstrom et al., 2017).

BA homeostasis is tightly regulated by enterohepatic signaling, whereas BA accumulation causes a series of diseases including inflammatory bowel disease, cholestatic hepatitis, primary biliary cirrhosis (Fiorucci et al., 2021). Clinical investigation reported that the serum level of BAs is relatively higher in NASH patients compared with healthy controls (Sydor et al., 2020), suggesting that the alteration of the BA pool in the development of NASH. Since IBAT is in charging of the efficient BA reabsorption, IBAT inhibitors that prevent BA accumulation are promising agents in improving NASH (Yamauchi et al., 2021).

Huazhi-Rougan (HZRG) formula is a Chinese patent drug designed according to the theories of Traditional Chinese medicine (TCM). Targeting the TCM pathogenesis of damp-heat of NAFLD, HZRG has been widely used to treat NAFLD and its complications. Previous studies demonstrated that HZRG treatment significantly improved the CT value, hyperlipidemia, and reduced serum ALT and AST levels in NAFLD patients (Wang et al., 2021a). However, the underlying mechanisms are largely unknown.

The present study aimed to examine the effects of HZRG on NASH mice. We demonstrated that 4-week HZRG treatment improved liver lipid accumulation, injury and inflammation in mice fed a methionine- and choline-deficient (MCD) diet. We further identified that HZRG enhanced fecal BA excretion *via* inhibiting IBAT, and the modulation of BA profiles, gut dysbiosis as well as the intestinal environment all contributed to the beneficial effects of HZRG on NASH mice.

## Materials and Methods

### Preparation of Huazhi-Rougan Granule

HZRG granule is a patent TCM drug, composed of 16 herbal or medicinal fungi species: *Artemisia scoparia* Waldst. & Kitam. (Yin-Chen), *Cassia abbreviata* Oliv. (Jue-Ming-Zi), *Rheum officinale* Baill. (Da-Huang), *Alisma orientale* (Sam.) Juz. (Ze-Xie), Polyporus umbellatus (Pers.) Fries. (Zhu-Ling), *Crataegus pinnatifida* Bunge. (Shan-Zha), *Atractylodes lancea* (Thunb.) DC. (Cang-Shu), *Atractylis macrocephala* (Koidz.) Hand. -Mazz. (Bai-Shu), *Citrus reticulata* Blanco (Chen-Pi), *Trichosanthes kirilowii* Maxim. (Gua-Lou), *Ligustrum lucidum* W. T. Aiton (Nv-Zhen-Zi), *Eclipta prostrata* (L.) L. (Mo-Han-Lian), *Lycium barbarum* L. (Gou-Qi-Zi), *Cirsium setosum* (Willd.) Besser (Xiao-Ji), *Bupleurum chinense* DC. (Chai-Hu), and *Glycyrrhiza uralensis* Fisch. (Gan-Cao). The ratio of each component in the formula was defined based on a previous study (Wang et al., 2021a). The granules were purchased from Shandong New Time Pharmaceutical CO., Ltd. The chemical constituents in HZRG granules were analyzed based on UPLC-Q-TOF/MS approach (Hu et al., 2019).

### Animal and Diet

Forty male C57BL/6J mice of 6-week age were purchased from Gempharmatech Experimental Animal Technology Co. Ltd. (Jiangsu, China), and placed in the specific-pathogen-free environment at constant temperature (22 ± 2°C) and humidity (55 ± 15%), and 24 h light/dark alternation. The mice were divided into four groups after 1-week acclimatization: control group (*n* = 10) received chow diet (Research Diet, C17040502), NASH group (*n* = 10) received MCD diet (Research Diet, A02082002B), HZRG high dose (HRH, 6 g/kg/d) and low dose (HRL, 3 g/kg/d) groups (*n* = 10 per group) received MCD diet plus HZRG administration. The low dose of HZRG was equivalent to the effective clinical dose, while the double-dose was defined as a high dose. The drugs were dissolved in 0.5% carboxymethyl cellulose sodium solution (CMC-Na) and administered to the mice by gavage (0.1 ml/10 g body weight) once a day for 4 weeks, the control and NASH mice were given equivalent 0.5% CMC-Na solution. At the end of the experiment, mice were anesthetized *via* 2% pentobarbital sodium injection (1.5 ml/kg). Blood was collected to separate serum for biological analysis. A portion of the liver was fixed in 4% paraformaldehyde solution. Intestine, cecal feces, and the rest of liver portions were snap-frozen in liquid nitrogen and then stored at −80°C refrigerator. All mice were received humane care during this experiment, and the experiment was approved by the Animal Experiment Ethics Committee of Gempharmatech CO., Ltd. IACUC (Approval number: GPTAP20200721-2).

### Liver Histopathology

Liver pathological alterations were presented by the established method of our lab (Li et al., 2021). In brief, liver tissues were fixed, then dehydrated and embedded in paraffin. Paraffin-embedded tissue was cut into 4 μm sections and stained with hematoxylin-eosin (H&E) according to the standard process (Kohypath, Shanghai, China). For Oil Red O (ORO) staining, frozen liver tissues were embedded in Tissue-Tek OCT Compound (Sakura, Tokyo, Japan), cut into ∼8 μm sections, and stained with ORO reagent (Sigma, St. Louis, MO, United States). For immunohistochemical (IHC) analysis, anti-F4/80 (70076 s, cell signaling technology) primary antibody, and biotinylated goat anti-rabbit IgG (BOSTER, SA1022) were applied. Images were captured under a Nikon Eclipse 50i microscope (Nikon, Tokyo, Japan) with a magnification of ×200.

### Analysis of Serum and Liver Biochemical Parameters

The serum alanine aminotransferase (ALT), aspartate aminotransferase (AST), and were analyzed by a TBA-40FR Fully Automatic Biochemical Analyzer (TOSHIBA, Japan) according to the manufacturer’s protocol. Serum tumor necrosis factor-alpha (TNF-α) was detected using a mouse ELISA kit (mlbio, Shanghai, China). The liver tissue in ethanol was homogenized to collect supernatant for detecting TC and TG contents in the liver with certain kits (Nanjing Jiancheng Bioengineering Institute).

### 16S rDNA Sequence

Cecal feces of mice were collected for 16S rDNA analysis of gut microbiota. Microbial genome DNA was extracted using the Qiagen QIAamp DNA Stool Mini Kit (Qiagen, Dusseldorf, Germany), and quantified and characterized by the NanoDrop 2000°C spectrophotometer and agarose gel electrophoresis, respectively. The V3-V4 region of the bacterial 16S ribosomal RNA was amplified by PCR and used for the following analysis. The sequencing and analysis were performed as previously reported (Shu et al., 2021).

### Bile Acid Profile Analysis

The BA profile of fecal and serum sample was quantified by ultra-performance liquid-chromatography coupled with triple quadrupole mass spectrometry (UPLC-TQMS, Waters, Milford, MA) according to the previous method (Shu et al., 2021).

### Western Blot

Liver and intestinal samples were homogenized in RIPA buffer added with protease and phosphatase inhibitors. Full centrifugation at low temperature (15 min at 12,000 g) to obtain supernatant, and the protein concentration was quantified by BCA kit (Epizyme, Shanghai, China), proteins electrophoresis using the 10% sodium dodecyl sulfate-polyacrylamide gel and transferred onto 0.45 μm PVDF membranes (Millipore, United States). Subsequently, the PVDF membranes were socked in 5% skim milk containing 140 mmol/L NaCl, 20 mmol/L Tris-HCl (pH 7.5), and 0.1% Tween 20°at room temperature for 60 min, and incubated with primary antibodies at 4°C overnight: FXR mouse monoclonal antibody (72105S, CST, United States), TGR5 rabbitpolyclonal antibody (72,608, Abcam, United States), ZO-1 (ab216880, Abcam, United States), Occludin (ab 216,327, Abcam, United States), Claudin 2 (ab53032, Abcam, United States), P-P65 rabbit monoclonal antibody (3031S, CST, United States), P65 rabbit monoclonal antibody (8242S, CST, United States), β-actin (Hua-an Biotech Inc., Hangzhou, China), and then incubated with horseradish peroxidase-conjugated secondary antibodies at room temperature for another 60 min. The protein bands were visualized by an ECL chemiluminescence detection kit (WBKLS0500, Millipore, United States) with an enhanced chemiluminescence system (Tanon 5200, Shanghai, China).

### Real-Time Quantitative PCR

Liver tissues were homogenized in TRIzol reagent (Invitrogen Corp, Carlsbad, CA, United States) and the total RNA was isolated. RNA concentration was measured using a NanoDrop 2000°C spectrophotometer, and was reversely transcribed into complementary DNA by reverse transcription kit (Accurate Biology, Shanghai, China). The PCR primers (Shanjin Biotech, Shanghai, China) showed in Table 1. GAPDH was used as the internal control, and the expression of the target gene was normalized to GAPDH expression, and the relative expression was calculated by the 2^−ΔΔT^ method.

**TABLE 1:** Sequences of the primers used for RT-qPCR.

Gene	Forward primer	Reverse primer
*Tnfα*	ACG​TGG​AAC​TGG​CAG​AAG​AG	GGT​TGT​CTT​TGA​GAT​CCA​TGC
*Il1β*	AAA​TGA​TGG​CTT​ATT​ACA​GTG​GC	CTT​GCT​GTA​GTG​GTG​GTC​GG
*Ibat*	ATG​GCG​ACA​TGG​ACC​TCA​G	TCC​CGA​GTC​AAC​CCA​CAT​C
*Ostα*	ACC​TCG​TTT​TAT​GCC​GTA​TGC	TCG​GGG​TGT​CCT​TCA​GTG​TC
*Ostβ*	CTG​CTG​GAA​GAA​ATG​CTT​TGG	TGG​TGT​TTC​TTT​GTC​TTG​TGG​C
*Mrp2*	TGC​GTC​TTT​TCC​TGG​ATT​ACC	GTG​ATG​TTG​AGG​GCG​TTG​G
*Mrp3*	AGC​CTA​AAC​ATT​CAA​ATC​CCG	CAG​AGC​CCT​TTA​CAG​ACA​CCA​C
*Gapdh*	GTGCCGCCTGGAGAAACC	GGT​GGA​AGA​GTG​GGA​GTT​GC

### Statistical Analysis

All the data are collected and expressed as mean ± standard deviation (SD). Statistical analysis was performed using a one-way analysis of variance (ANOVA). Independent-sample *t* test was used to compare differences between two groups. Mann-Whitney U tests and Spearman correlation were performed using SPSS 26.0 software. *p* < 0.05 was considered of statistical significance.

## Results

### Chemical Profiling of Huazhi-Rougan

**FIGURE 1:**
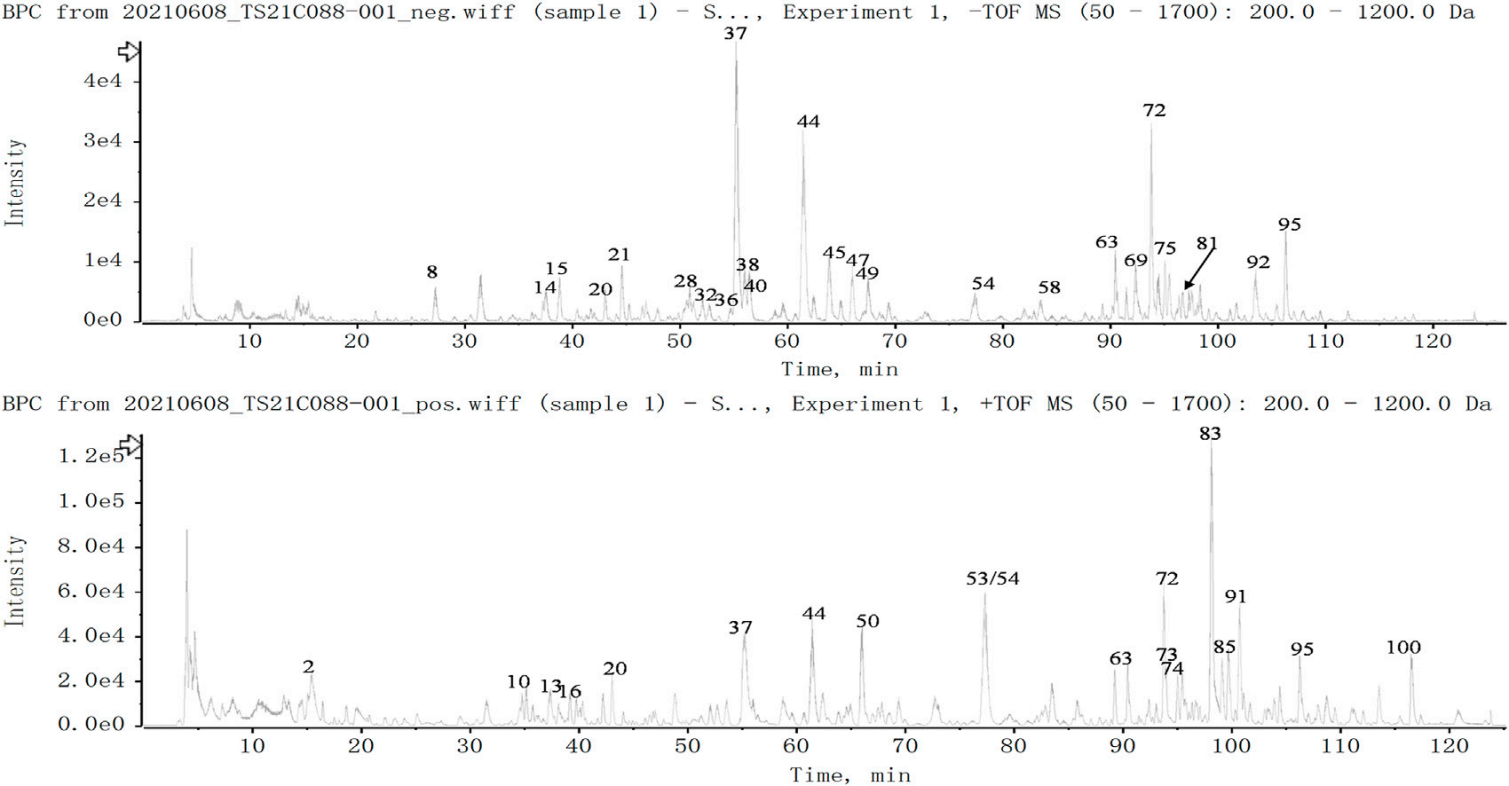
Total ion chromatogram of constituents in HZRG. Agilent 1290 UPLC system was applied to analyze the chemical profiling of HZRG granules, data were collected under both negative ion mode and positive ion mode, and processed by Analyst Ver. 1.6 software.

A total of 100 constituents have been identified or tentatively characterized in HZRG granule (compounds of 16 medicinal) under positive or negative ion mode (Figure 1 and Table 2). Among them, 20 constituents attributed to *Lycium barbarum* L. (Gou-Qi-Zi)*,* 13 attributed to *Eclipta prostrata* (L.) L. (Mo-Han-Lian)*,* 11 attributed to *Ligustrum lucidum* W. T. Aiton (Nv-Zhen-Zi), six attributed to *Atractylodes lancea* (Thunb.) DC. (Cang-Shu), 8 attributed to *Rheum officinale* Baill. (Da-Huang)*,* 11 attributed to *Citrus reticulata* Blanco (Chen-Pi)*,* eight attributed to *Glycyrrhiza uralensis* Fisch. (Gan-Cao)*,* three attributed to *Crataegus pinnatifida* Bunge. (Shan-Zha), five attributed to *Bupleurum chinense* DC. (Chai-Hu)*,* four attributed to *Trichosanthes kirilowii* Maxim. (Gua-Lou)*,* four attributed to *Polyporus umbellatus* (Pers.) Fries. (Zhu-Ling), three attributed to *Artemisia scoparia* Waldst. and Kitam. (Yin-Chen)*,* three attributed to *Atractylis macrocephala* (Koidz.) Hand. -Mazz. (Bai-Shu)*,* and two was attributed to *Cirsium setosum* (Willd.) Besser (Xiao-Ji) (Table 2). Collectively, the dominant constituents in HZRG granule belong to flavonoids, alkaloids and lactones.

**TABLE 2:** The detected ion chromatogram of constituents in HZRG.

NO	T_R_(min)	Selected ion	*m/z* Measured	*m/z* calculated	ppm	Formula	Identification	MS/MS fragmentation	Attribution
1	8.83	[M-H]^−^	337.0784	337.0776	2.3	C_12_H_18_O_11_	2-O-β-D-glucopyranosyl L-ascorbic acid	337.0750; 277.0545; 174.0150	Gou-Qi-Zi
2	15.40	[M + H]^+^	294.1528	294.1547	−6.9	C_12_H_23_NO_7_	Fructoseleucine	276.1426; 258.1318; 230.1369; 212.1262; 182.1154	Gou-Qi-Zi
3	16.43	[M + H]^+^	268.1026	268.104	−5.3	C_10_H_13_N_5_O_4_	Adenosine	136.0601; 119.0329	/
4	17.22	[M + H]^+^	284.0963	284.0989	−9.3	C_10_H_13_N_5_O_5_	Guanosine	152.0556; 135.0285; 110.0329	/
5	19.63	[M + H]^+^	328.1373	328.1391	−5.4	C_15_H_21_NO_7_	Fructosephenylalanine	310.1261; 292.1160; 264.1206; 192.0996; 120.784	Gou-Qi-Zi
6	19.77	[M-H]^−^	493.1212	493.1199	2.6	C_19_H_26_O_15_	Galloylsucrose	493.1182; 403.0838; 283.0438; 169.0136	/
7	21.7	[M-H]^−^	315.0723	315.0722	0.5	C_13_H_16_O_9_	Protocatechuic acid-3-O-glucoside	315.0727; 153.0114; 153.0187; 108.0213	Gou-Qi-Zi
8	27.3	[M-H]^−^	353.0878	353.0878	−4.3	C_16_H_18_O_9_	Neochlorogenic acid	353.0850; 191.0557; 179.0343; 135.0447	Mo-Han-Lian, Yin-Chen, Gou-Qi-Zi, Cang Zhu, Shan Zha
9	34.77	[M + H]^+^	798.3654	798.3655	−0.1	C_37_H_55_N_3_O_16_	N (1),N (8)-bis-dihydrocaffeoyl -spermidine-di-hexoside	798.3659; 636.3117; 474.2589; 384.1634; 222.1109	Gou-Qi-Zi
10	35.16	[M + H]^+^	796.3467	796.344	3.4	C_44_H_49_N_3_O_11_	N (1)-dihydrocaffeoyl-N (8)-caffeoyl-spermidine-di-hexoside	796.3512; 634.3006; 472.2430; 382.1492; 220.0978	Gou-Qi-Zi
11	37.23	[M + H]^+^	796.3513	796.3499	1.8	C_37_H_53_N_3_O_16_	N (1)-caffeoyl-N (8)-dihydrocaffeoyl-spermidine-di-hexoside	796.3538; 634.3020; 472.2546; 382.1498; 220.0973	Gou-Qi-Zi
12	37.25	[M-H]^−^	389.1083	389.1089	−1.6	C_16_H_22_O_11_	Secologanoside	389.1109; 345.1207; 209.0461; 165.0562	Nv-Zhen-Zi
13	37.36	[M + H]^+^	636.3106	636.3127	−3.3	C_31_H_45_N_3_O_11_	N (1),N (8)-bis-dihydrocaffeoyl-spermidine-hexoside	636.3134; 474.2596; 384.1646; 222.1116	Gou-Qi-Zi
14	37.5	[M-H]^−^	353.0881	353.0878	0.8	C_16_H_18_O_9_	Chlorogenic acid	191.0550; 173.0444; 161.0259	Mo-Han-Lian, Yin-Chen, Gou-Qi-Zi, Cang Zhu, Shan Zha
15	38.75	[M-H]^−^	353.0893	353.0878	4.2	C_16_H_18_O_9_	Cryptochlorogenic acid	191.0554; 173.0454	Mo-Han-Lian, Yin-Chen, Gou-Qi-Zi, Cang Zhu, Shan Zha
16	39.15	[M + H]^+^	634.2986	634.297	2.5	C_31_H_43_N_3_O_11_	N (1)-dihydrocaffeoyl-N8-caffeoyl-spermidine-hexoside	634.2955; 472.2446; 310.2116; 220.0951	Gou-Qi-Zi
17	39.73	[M + H]^+^	634.2978	634.297	1.2	C_31_H_43_N_3_O_11_	N (1)-caffeoyl-N8-dihydrocaffeoyl-spermidine-hexoside	634.2982; 472.2419; 382.1487; 220.0964	Gou-Qi-Zi
18	40.34	[M + H]^+^	474.2580	474.2599	−3.9	C_25_H_35_N_3_O_6_	N (1),N (8)-bis-(dihydrocaffeoyl)spermidine	474.2580; 222.1110	Gou-Qi-Zi
19	42.2	[M + H]^+^	472.2417	472.2442	−5.3	C_25_H_33_N_3_O_6_	N (1)-caffeoyl-N (8)-dhydrocaffeoylspermidine	472.2436; 310.2105; 220.0947; 163.0373	Gou-Qi-Zi
20	43.02	[M-H]^−^	593.1517	593.1512	0.2	C_27_H_30_O_15_	Vicenin-II	593.1486; 473.1085; 353.0643	Gan-Cao, Chen-Pi
21	44.62	[M-H]^−^	515.1189	515.1195	−1.2	C_25_H_24_O_12_	1,​3-​Dicaffeoylquinic acid	515.1224; 353.0869; 191.0555; 179.0351; 135.0449	Mo-Han-Lian, Gou-Qi-Zi, Cang Zhu
22	45.21	[M-H]^−^	785.2514	785.251	0.5	C_35_H_46_O_20_	Echinacoside	785.2558; 623.2188; 161.0250	Nv-Zhen-Zi
23	45.52	[M-H]^-^	367.1027	367.1035	−2.1	C_17_H_20_O_9_	3-O-feruloylquinic acid	191.0569; 173.0449	Gou-Qi-Zi
24	46.82	[M + FA-H]^-^	621.2769	621.2764	0.8	C_27_H_44_O_13_	Atractyloside I	621.2749; 575.2742; 413.2193	Cang-Zhu
25	46.99	[M-H]^−^	479.0839	479.0831	1.6	C_21_H_20_O_13_	Myricetin-3-O-β-galactoside	479.0852; 317.0285	/
26	49.83	[M-H]^−^	701.2333	701.2298	4.9	C_31_H_42_O_18_	Neonuezhenide	701.2314; 469.1386; 315.1095	Nv-Zhen-Zi
27	50.33	[M-H]^−^	609.1477	609.1461	2.6	C_27_H_30_O_16_	Rutin	609.1468; 301.0339; 300.0265	Shan-Zha, Xiao-Ji
28	50.67	[M-H]^−^	549.1628	549.1614	2.6	C_26_H_30_O_13_	Liquiritinapioside	549.1632; 417.1193; 255.069	Gan-Cao
29	51.23	[M-H]^-^	417.1192	417.1191	0	C_21_H_22_O_9_	Liquiritin	417.1165; 255.0654; 135.0077	Gan-Cao
30	52.02	[M-H]^−^	623.1970	623.1981	−1.8	C_29_H_36_O_15_	Verbascoside	623.1962; 461.1615; 161.0232	Nv-Zhen-Zi
31	52.07	[M-H]^−^	463.0897	463.0882	3.2	C_21_H_20_O_12_	Hyperoside	463.0879; 301.0323; 300.0265; 271.0221	Shan-Zha
32	52.14	[M-H]^−^	685.2351	685.2349	0.3	C_31_H_42_O_17_	Nuezhenide	685.2334; 523.1779; 453.1365; 299.1118	Nv-Zhen-Zi
33	52.66	[M-H]^−^	463.0891	463.0882	1.9	C_21_H_20_O_12_	Isoquercitrin	463.0869; 301.0339; 300.0243	Shan-Zha
34	52.67	[M-H]^−^	447.0936	447.0933	0.7	C_21_H_20_O_11_	Astragalin	447.0928; 285.0380; 284.0322	Nv-Zhen-Zi
35	53.85	[M + H]^+^	197.1161	197.1172	−5.7	C_11_H_16_O_3_	Loliolide	197.1170; 179.1055; 161.0946; 133.0996; 105.0690	Gua-Lou
36	54.78	[M-H]^−^	378.9746	378.9765	−5.1	C_15_H_8_O_10_S	1,8,9-Trihydroxy-3-(sulfooxy)-6H-benzofuro [3,2-c][1]benzopyran-6-one	378.9712; 299.0173; 255.0266; 211.0405	Mo-Han-Lian
37	55.22	[M-H]^−^	685.2348	685.2349	−0.2	C_31_H_42_O_17_	Specnuezhenide	685.2364; 523.1821; 453.1392; 421.1495; 299.1112	Nv-Zhen-Zi
38	56	[M-H]^−^	579.1705	579.1719	−2.5	C_27_H_32_O_14_	Naringin	579.1682; 271.0605; 151.0037	Chen-Pi
39	56.15	[M-H]^−^	623.2003	623.1981	3.5	C_29_H_36_O_15_	Isoverbascoside	623.1989; 461.1692; 315.1106; 161.0231	Nv-Zhen-Zi
40	56.41	[M-H]^−^	515.1197	515.1195	0.4	C_25_H_24_O_12_	Isochlorogenic acid B	515.1207; 353.0873; 191.0557; 137.0449; 179.0345	Mo-Han-Lian/Gou-Qi-Zi/Cang-Shu
41	59.55	[M-H]^−^	515.1196	515.1195	0.2	C_25_H_24_O_12_	Isochlorogenic acid A	515.1175; 353.0867; 191.0560; 179.0349	Mo-Han-Lian/Gou-Qi-Zi/Cang-Shu
42	60.78	[M-H]^−^	919.2716	919.2725	−1	C_39_H_52_O_25_	Cassiaside B2	919.2707; 647.2037; 545.1625; 271.0606; 256.0383	Jue-Ming-Zi
43	61.43	[M-H]^−^	901.2645	901.2619	2.9	C_39_H_50_O_24_	Chrysophanol-1-O-β-D-glucopyranosyl-(1→3)-O-β-D-glucopyranosyl-(1→6)-O-β-D-glucopyranosyl-(1→6)-O-β-D-glucopyranoside	901.2654; 647.2058; 545.1692; 253.0500	Jue-Ming-Zi
44	61.5	[M-H]^−^	609.1828	609.1825	0.5	C_28_H_34_O_15_	Hesperidin/Neohesperidin	609.1810; 301.0706; 286.0471	Chen-Pi
45	63.87	[M-H]^−^	515.1197	515.1195	0.4	C_25_H_24_O_12_	Isochlorogenic acid C	515.1209; 353.0875; 191.0556; 173.0458; 179.0347	Mo-Han-Lian/Gou-Qi-Zi/Cang-Shu
46	64.98	[M-H]^−^	739.2090	739.2091	−0.1	C_33_H_40_O_19_	Chrysophanol 1-triglucoside	739.2048; 485.1503; 253.0491	Da-Huang/Jue-Ming-Zi
47	66.01	[M-H]^-^	595.1654	595.1668	−2.4	C_27_H_32_O_15_	Toralactone 9-gentiobioside	595.1644; 271.0598; 256.0364	Jue-Ming-Zi
48	67.11	[M-H]^−^	539.1787	539.177	3.1	C_25_H_32_O_13_	Oleuropein	539.1797; 377.1249; 275.0904	Nv-Zhen-Zi
49	67.45	[M-H]^-^	491.1189	491.1195	−1.2	C_23_H_24_O_12_	Aurantio-obtusin-beta-D-glucoside	491.1203; 476.0939; 461.0657; 313.0344	Jue-Ming-Zi
50	69.42	[M-H]^−^	595.1658	595.1668	−1.8	C_27_H_32_O_15_	Rubrofusarin 6-gentiobioside	595.1732; 271.0617; 256.0372	Jue-Ming-Zi
51	69.98	[M-H]^-^	417.1205	417.1191	3.3	C_21_H_22_O_9_	Isoliquiritin	417.1173; 255.0684	Gan-Cao
52	72.96	[M-H]^−^	565.1583	565.1563	3.6	C_26_H_30_O_14_	Cassiaside B	567.1592; 271.0608; 256.0388	Jue-Ming-Zi
53	77.18	[M-H]^−^	591.1755	591.1719	6	C_28_H_32_O_14_	Linarin	313.0628; 283.0627; 268.0374	Xiao-Ji/Mo-Han-Lian
54	77.41	[M-H]^−^	1071.3565	1071.3562	0.3	C_48_H_64_O_27_	Oleonuezhenide	1071.3606; 909.3632; 771.2355; 685.2372; 523.1854; 453.1389; 299.1130	Nv-Zhen-Zi
55	79.22	[M + H]^+^	309.0864	309.087	−1.9	C_17_H_12_N_2_O_4_	Flazin	291.0755; 263.0806; 206.0832; 205.0750	Gua-Lou
56	81.97	[M-H]^−^	431.1001	431.0984	4	C_21_H_20_O_10_	Aloe-emodin-8-O-glucoside	431.0938; 268.0364; 240.0412	Da-Huang
57	82.89	[M-H]^−^	1071.3643	1071.3562	7.5	C_48_H_64_O_27_	Nuezhenoside G13	1071.3577; 909.2898; 685.2326; 523.1806; 453.1380	Nv-Zhen-Zi
58	83.55	[M-H]^−^	431.0999	431.0984	3.5	C_21_H_20_O_10_	Emodin-8-O-glucoside	431.0976; 269.0446; 225.0544	Da-Huang
59	84.45	[M-H]^−^	313.0355	313.0354	0.4	C_16_H_10_O_7_	Wedelolactone	313.0340; 298.0102; 269.0067	Mo-Han-Lian
60	86.3	[M + H]^+^	728.4003	728.3978	3.5	C_36_H_53_N_7_O_9_	Citrusin Ⅲ	728.3985; 700.4058; 587.3143; 474.2357	Chen-Pi
61	87.68	[M-H]^−^	313.0343	313.0354	−3.4	C_16_H_10_O_7_	Laccaic acid D	313.0335; 269.0438; 241.0527; 226.0273	Da-Huang
62	88.43	[M + H]^+^	477.3209	477.3211	−0.3	C_28_H_44_O_6_	Polyporusterone B	477.3249; 459.3132; 441.3084; 423.2857; 357.2080	Zhu-Ling
63	90.46	[M + FA-H]^−^	841.4624	841.4591	3.9	C_42_H_68_O_14_	Eclalbasaponin C	841.4601; 795.4505; 675.4136; 633.3990	Mo-Han-Lian
64	90.52	[M + H]^+^	479.3379	479.3367	2.5	C_28_H_46_O_6_	Polyporusterone A	479.3353; 461.3243; 443.3103; 425.3071	Zhu-Ling
65	90.63	[M-H]^−^	837.3933	837.3914	2.2	C_42_H_62_O_17_	Licorice saponin G2	837.3879; 351.0558	Gan-Cao
66	91.49	[M-H]^−^	875.4100	875.4104	−0.5	C_42_H_68_O_17_S	Eclalbasaponinn VI	875.4086; 713.3535	Mo-Han-Lian
67	92.31	[M + FA-H]^−^	973.5361	973.5378	−1.7	C_48_H_80_O_17_	Saikosaponin f	973.5380; 927.5314; 781.4683	Chai-Hu
68	92.38	[M-H]^−^	837.3916	837.3914	0.2	C_42_H_62_O_17_	Licorice saponin Q2	837.3922; 351.0539	Gan-Cao
69	92.43	[M-H]^−^	329.2321	329.2333	−3.8	C_18_H_34_O_5_	Tianshic acid	329.2324; 229.1442; 211.1327; 183.1390; 171.1023	Gua-Lou
70	92.84	[M + FA-H]^−^	971.5221	971.5221	0	C_48_H_78_O_17_	Saikosaponin C	971.5298; 925.5173; 779.4491	Chai-Hu
71	93.06	[M + H]^+^	373.1279	373.1282	−0.7	C_20_H_20_O_7_	Isosinensetin	373.1273; 358.1037; 343.0798; 327.0469; 315.0846	Chen-Pi
72	93.76	[M-H]^−^	821.3955	821.3965	−1.2	C_42_H_62_O_16_	Glycyrrhizic acid	821.3917; 351.0547	Gan-Cao
73	93.95	[M + H]^+^	505.3522	505.3524	−0.3	C_30_H_48_O_6_	16-oxoalisol A	505.3533; 487.3406; 469.3321; 451.3202; 415.2838	Ze-Xie
74	95.05	[M + FA-H]^−^	825.4663	825.4642	2.5	C_42_H_68_O_13_	Saikosaponin a	825.4712; 779.4602; 617.4064	Chai-Hu
75	95.06	[M-H]^-^	821.3985	821.3965	5.4	C_42_H_62_O_16_	Licoricesaponin K2	821.3906; 351.0533	Gan-Cao
76	95.15	[M + H-H2O]^+^	529.3507	529.3524	−2.6	C_32_H_50_O_7_	23-Acetyl 16-oxoalisol A	529.3515; 469.3311; 451.3193	Ze-Xie
77	95.28	[M-H]^−^	299.0550	299.0561	−3.7	C_16_H_12_O_6_	Chrysoeriol	299.0549; 284.0293; 256.0356	Gua-Lou
78	95.46	[M-H]^−^	329.0673	329.0667	1.9	C_17_H_14_O_7_	Aurantio-obtusin	329.0648; 314.0405; 299.0156; 271.0213; 243.0268	Jue-Ming-Zi
79	95.67	[M + H]^+^	373.1286	373.1282	1.1	C_20_H_20_O_7_	Sinensetin	373.1273; 357.0950; 343.0785; 329.0995; 312.0967	Chen-Pi
80	96.38	[M + FA-H]^−^	825.4676	825.4642	4.1	C_42_H_68_O_13_	Saikosaponin d	825.4818; 779.4634; 617.4057	Chai-Hu
81	96.7	[M-H]^−^	633.4017	633.4008	1.4	C_36_H_58_O_9_	Ecliptasaponin D	633.3991; 587.3928; 161.0441	Mo-Han-Lian
82	97.58	[M-H]^−^	283.0254	283.0248	2.1	C_15_H_8_O_6_	Rhein	283.0222; 239.0324; 211.0381; 183.0422	Da-Huang/Jue-Ming-Zi
83	98.09	[M + H]^+^	403.1369	403.1387	−4.6	C_21_H_22_O_8_	Nobiletin	403.1395; 388.1163; 373.0913; 355.0816	Chen-Pi
84	98.24	[M + FA-H]^−^	867.4801	867.4748	6.2	C_44_H_70_O_14_	3″-O-Acetylsaikosaponin a	867.4818; 821.0471; 779.4587; 617.4067	Chai-Hu
85	99.74	[M + H]^+^	433.1472	433.1493	−4.9	C_22_H_24_O_9_	3′,4′,3,5,6,7,8-Heptamethoxyflavone	433.1506; 418.1273; 403.1035	Chen-Pi
86	99.11	[M-H]^−^	357.0986	357.098	1.7	C_19_H_18_O_7_	Chrysoobtusin	357.0974; 342.0733; 327.0492; 312.0254; 284.0296	Jue-Ming-Zi
87	99.84	[M + H]^+^	487.3413	487.3418	−1	C_30_H_46_O_5_	Alisol C	487.3464; 469.3333; 451.3233; 397.2749	Ze-Xie
88	100.33	[M + H]^+^	249.1482	249.1485	−1.3	C_15_H_20_O_3_	AtractylenolideⅢ	231.1368; 203.1423; 189.0903; 163.0747; 149.0582	Bai-Shu/Cang-Shu
89	101.14	[M-H]^−^	343.0830	343.0823	2	C_18_H_16_O_7_	Obtusin	343.0820; 328.0580; 313.0339; 285.0377	Jue-Ming-Zi
90	101.71	[M-H]^−^	283.0612	283.0612	1.8	C_16_H_12_O_5_	Obtusifolin	283.0609; 268.0371; 240.0416; 239.0334	Jue-Ming-Zi
91	100.69	[M + H]^+^	373.1282	373.1282	0.1	C_20_H_20_O_7_	Tangeretin	373.1292; 358.1056; 343.0806	Chen-Pi
92	103.48	[M-H]^−^	269.0462	269.0455	2.4	C_15_H_10_O_5_	Emodin	269.0449; 241.0482; 225.0544	Da-Huang/Jue-Ming-Zi/Zhu-Ling
93	103.92	[M + H]^+^	529.3540	529.3524	2.9	C_32_H_48_O_6_	23-Acetyl alisol C	569.3588; 469.3347; 451.3241; 433.3152	Ze-Xie
94	105.86	[M + H]^+^	233.1524	233.1536	−5.2	C_15_H_20_O_2_	AtractylenolideⅡ	233.1525; 215.1422; 187.1459; 151.0739; 131.0836	Bai-Shu/Cang-Shu
95	106.28	[M + FA-H]^−^	535.3663	535.364	4.2	C_30_H_50_O_5_	Alisol A	535.3638; 489.3587; 471.3460; 339.2671	Ze-Xie
96	107.04	[M + H]^+^	515.3723	515.3731	−1.6	C_32_H_50_O_5_	23-Acetyl alisol B	515.3677; 497.3630; 455.3520; 437.3419; 419.3314	Ze-Xie
97	109.33	[M + H]^+^	231.1381	231.138	0.6	C_15_H_18_O_2_	Atractylenolide Ⅰ	231.1381; 185.1321; 155.0869; 143.0841	Bai-Shu/Cang-Shu
98	109.49	[M + H]^+^	515.3718	515.3731	−2.5	C_32_H_50_O_5_	Alisol B 11-monoacetate	515.3781; 497.3652; 419.336; 383.29558; 365.2857	Ze-Xie
99	114.28	[M + H]^+^	527.3736	527.3731	0.9	C_33_H_50_O_5_	Dehydropachymic acid	527.3689; 509.3714; 467.3473; 449.3215	Zhu-Ling
100	116.53	[M + H]^+^	515.3741	515.3731	2.1	C_32_H_50_O_5_	23-Acetyl alisol B Isomer	515.3773; 437.3437; 419.3320; 357.2828; 339.2677	Ze-Xie

### Huazhi-Rougan Attenuates Non-Alcoholic Steatohepatitis in Methionine-Choline-Deficiency Mice

**FIGURE 2:**
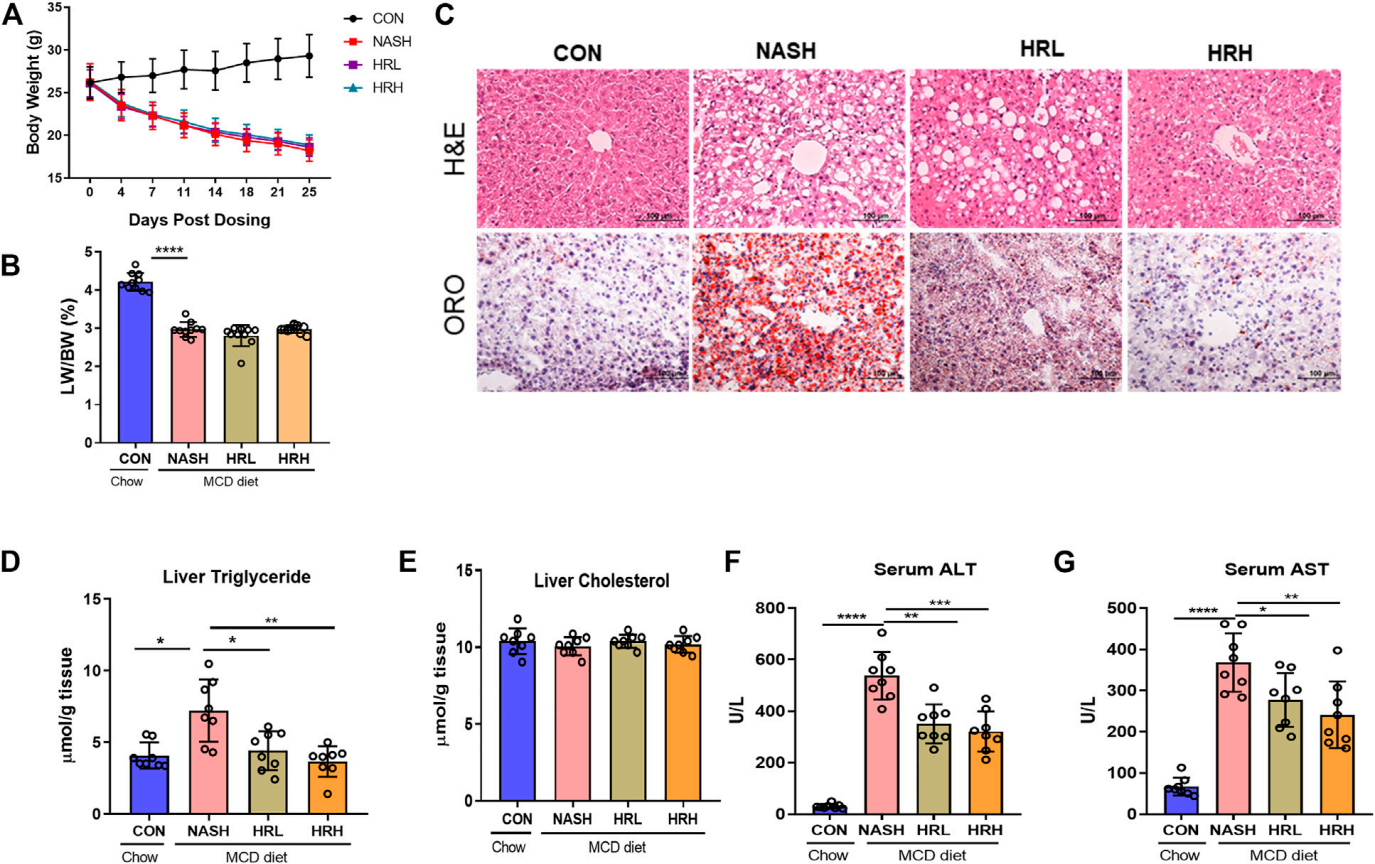
Effects of HZRG on MCD induced NASH mice. **(A)** Body weight; **(B)** liver/body weight ratio; **(C)** Representative H&E and ORO staining images of liver sections (magnification ×200); **(D,E)** Liver TG and TC content; **(F,G)** Serum ALT and AST levels. Data are expressed as mean ± SD, 10 animals were allocated for each group. ^*^ *p* < 0.05, ^**^ *p* < 0.01; ^***^ *p* < 0.001 between groups.

To investigate whether HZRG has an effect on NASH, we used MCD-fed mice as a NASH model. MCD-fed mice were treated with either low dose, high dose of HZRG, or vehicle for 4 weeks. MCD-fed mice showed a significant decrease in body weight and the liver/body weight ratio in comparison to control mice, but no statistical difference was found among treated groups (Figures 2A,B). MCD feeding induced obvious steatosis, inflammatory cell infiltration in liver sections as evidenced by H&E staining and ORO staining, and both high and low dose HZRG treatment significantly improved liver steatosis and reduced the infiltration of inflammatory cells (Figure 2C). The quantification of hepatic lipids revealed that HZRG treatment also decreased liver TG content, which was consistent with the pathological change (Figure 2D). However, the liver TC content showed no statistical difference among groups (Figure 2E). HZRG also significantly decreased serum ALT and AST levels in MCD-fed mice (Figures 2F,G), indicating the protective effects against liver damage. Collectively, these results suggest that HZRG attenuates NASH in MCD-fed mice, and the high dose was superior to the low dose HZRG.

### Huazhi-Rougan Alleviates Methionine-Choline-Deficiency-Induced Gut Dysbiosis

**FIGURE 3:**
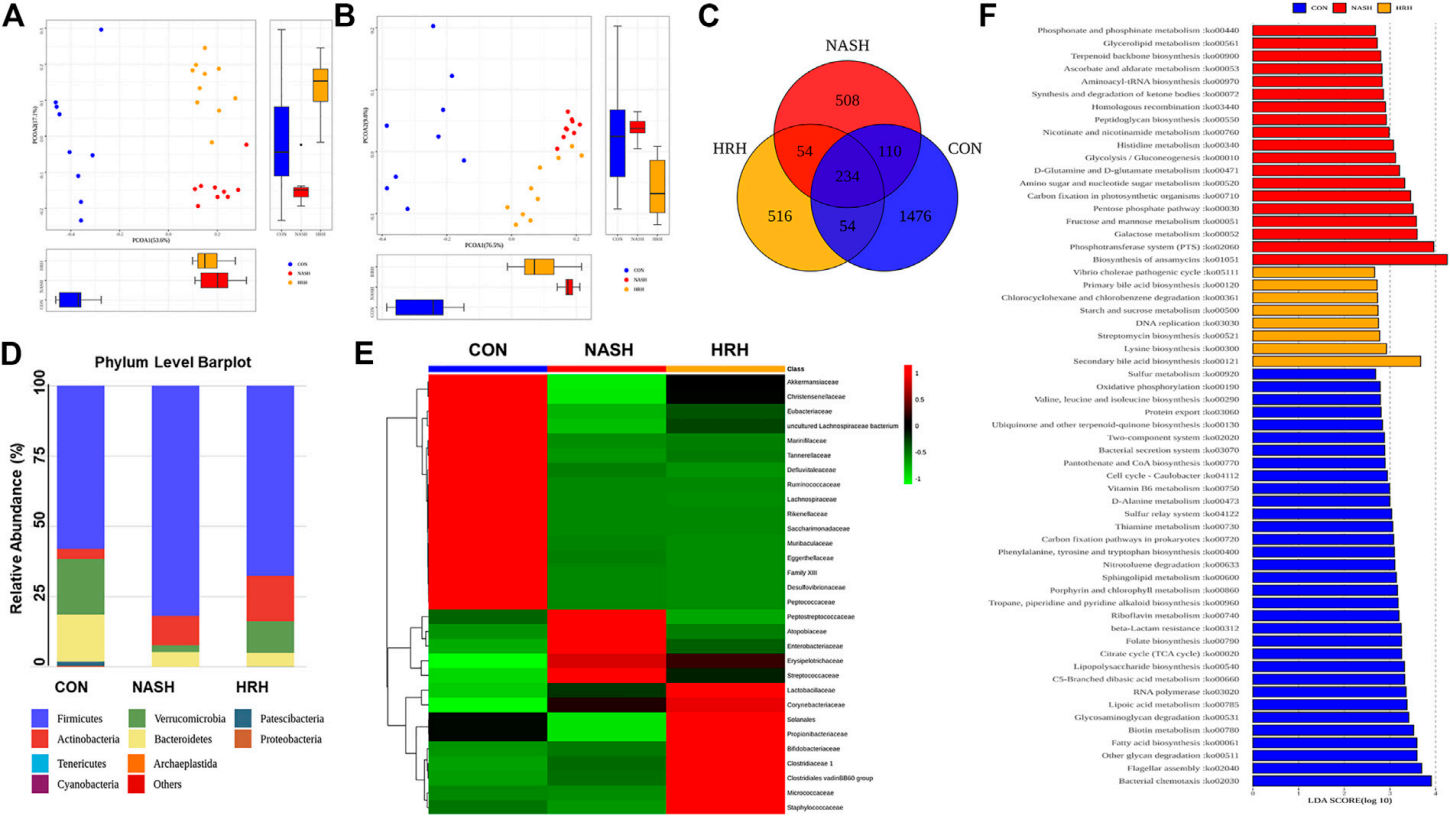
HZRG modulates gut microbiota in NASH mice. **(A)** PCoA analysis based on the weighted UniFrac analysis of OTUs; **(B)** PCoA analysis based on the unweighted UniFrac analysis of OTUs; **(C)** Venn Diagram; **(D)** Mean Phylum abundance of OTUs; **(E)** Mean family abundance of OTUs; **(F)** Pathways that based on PICRUST functional prediction. Data are expressed as mean ± SD of 9 or 10 animals per group.

Gut dysbiosis plays a pivotal role in the development and progression of NASH, and modulation of gut microbiota is a potential therapeutic strategy for NASH. HZRG contains various phytochemicals, such as flavonoids and alkaloids, which are known to regulate dysbiosis. We examined the effects of HZRG on the structure of gut microbiota by performing bacterial 16S rDNA sequence in feces, and observed a distinct clustering of microbiota for control, NASH, and HZH treatment groups using weighted (Figure 3A) and unweighted (Figure 3B) UniFrac-based principal coordinates analysis (PCoA), respectively. The comparison among the three groups revealed 1874 operational Taxonomic Units (OTUs) in the control group, 906 OTUs in the NASH group, and 858 OTUs in the HZRG-treated group, and a total of 234 OTUs shared all the samples (Figure 3C). At the phylum level, *Firmicutes*, *Bacteroidetes*, *Actinobactiria*, and *Verrucomicrobia* were the dominant four phyla (Figure 3D). The Family-level analysis revealed that the MCD diet increased the relative abundance of *Peptostreptococcaceae*, *Atopobiaceae*, *Enterobacteriaceae*, *Erysipelotrichaceae*, and *Streptococcaceae*, whereas HZRG decreased the relative abundance of these microbiomes. Notably, HZRG specifically increased the enrichment of *Lactobacillaceae*, *Bifidobacteriaceae*, *Clostruduaceae*, *Chostridiales VadinBB60*, *Corynebacteriaceae*, *Solanales*, *Propionibacteriaceae*, *Micrococcaceae*, and *Satphylococcaceae* (Figure 3E). In addition, the comparison was also conducted in genus and species levels (Supplementary Figure S1) Meanwhile, the functional prediction analysis based on PICRUST (phylogenetic investigation of communities by reconstruction of unobserved states) suggested that BA biosynthesis was the most obvious pathway upon HZRG treatment (Figure 3F). These results indicated that HZRG modulates the gut microbiota of NASH mice, resulting in the alleviation of dysbiosis in MCD-fed mice.

### Huazhi-Rougan Enhances Fecal Bile Acid Excretion

**FIGURE 4:**
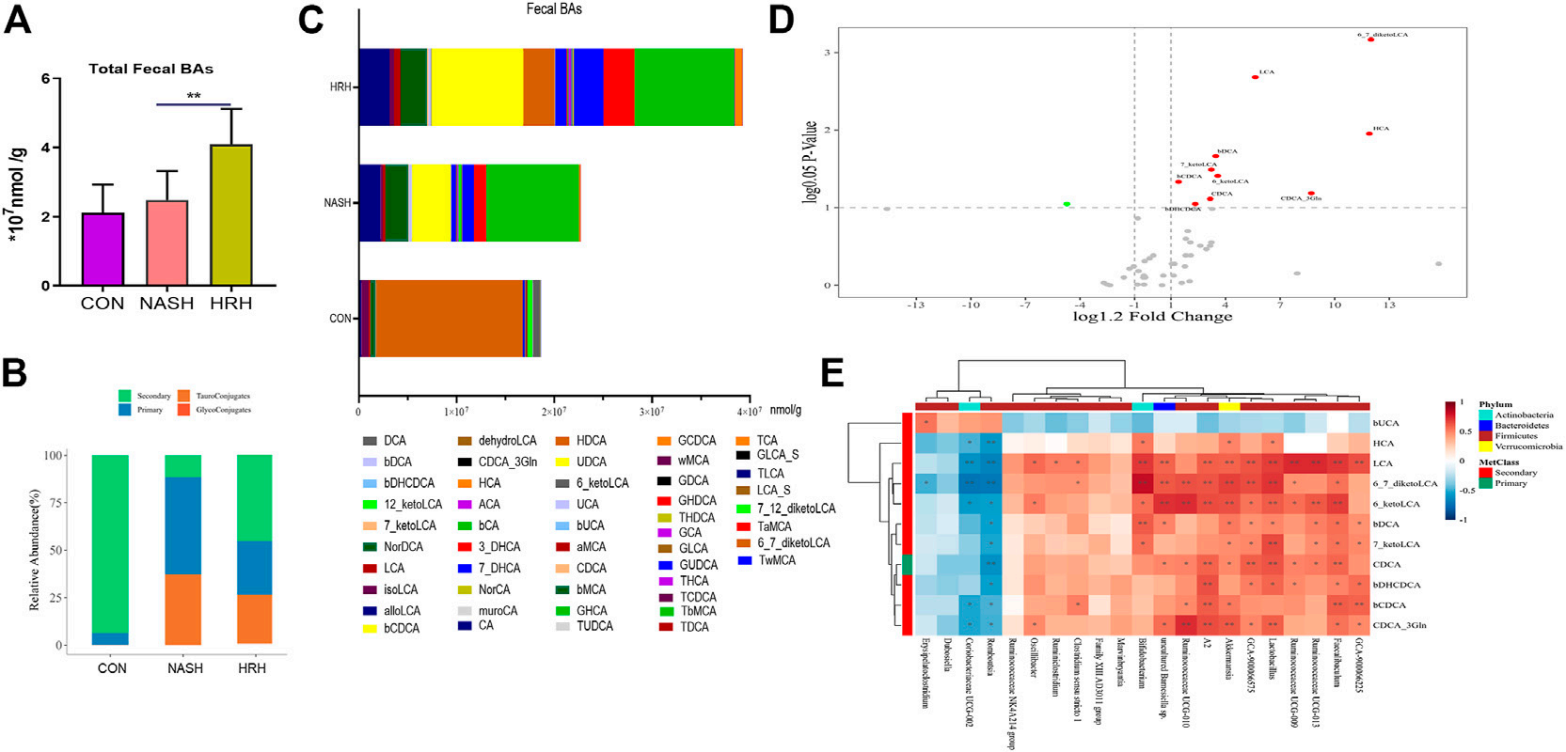
HZRG enhances fecal BA excretion. **(A)** The total fecal BA concentration; **(B)** The alteration of BA classification; **(C)** Fecal BA profile; **(D)** Volcano plot shows the change of BA species upon HZRG intervention (Red-increase, green-decrease); **(E)** Spearman correlation analysis of the relative abundance of microbial species and the BAs. Data are expressed as mean ± SD, *n* = 10 per group. ^*^ *p* < 0.05, ^**^ *p* < 0.01between groups.

To assess BA profile alternation in response to the of gut microbiota, a UPLC/TQMS based targeted metabolomics approach was applied to analyze the fecal BAs in mice. The results revealed that the total level of fecal BAs was dramatically elevated upon HZRG treatment in NASH mice, although the total BA level between control mice and NASH mice was not statistically different (Figure 4A). By analyzing BA composition, we found that the relative abundance of secondary BAs was significantly decreased in NASH mice, whereas HZRG intervention increased the percent of fecal secondary BAs (Figure 4B). The BA profiling showed that HZRG treatment significantly increased the secondary BA species such as LCA, ketoLCAs (6,7-keto, 6-keto, 7-keto), HCA and βDCA in NASH mice (Figures 4C,D). The changes in total fecal BA level along with the increased content of secondary BAs suggested that HZRG promoted fecal BA excretion, especially the secondary BA species. Transformation into secondary BAs largely depends on the action of gut microbiota. To explore the correlation of HZRG modulated fecal BAs and gut microbiota, a Spearman correlation was conducted between the relative abundance of the 21 differential microbial species and the 11 BA species in the NASH and HZRG groups. All of the 11 BAs had at least one significant correlation with a microbe (Figure 4E). βUCA was negatively correlated with microbial Genus *Erysipelatoclostridium*, *Dubosiella*, *Coriobacteriaceae UCG-002* and *Romboutsia*, whereas all the other differentiate BAs (DCA, HCA, LCA, ketoLCAs, *etc*) were positively correlated with these microbial species.

### Huazhi-Rougan Inhibits Bile Acid Reabsorption

**FIGURE 5:**
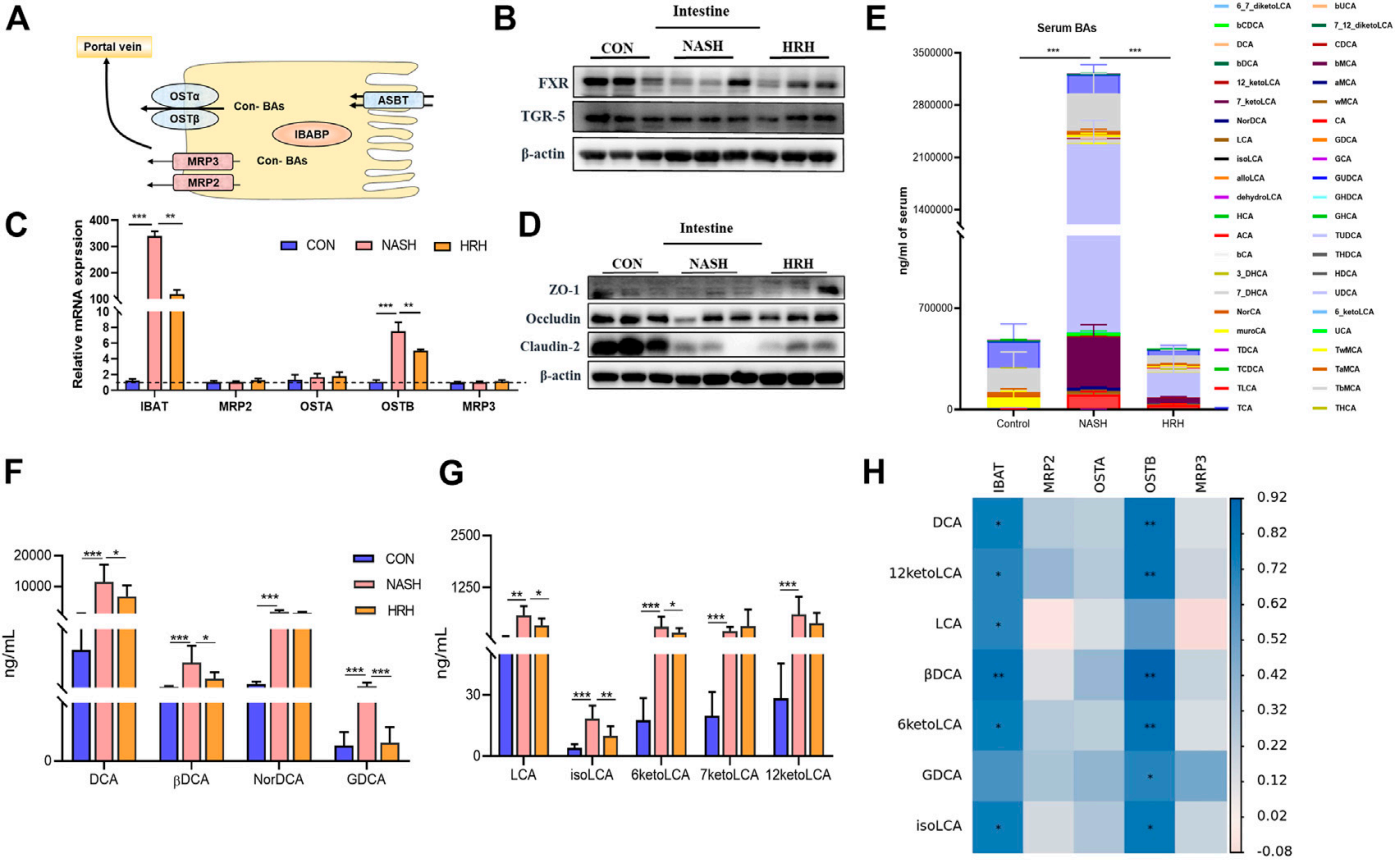
HZRG inhibits BA reabsorption. **(A)** The model of BA transport in the intestine; **(B)** The protein expression of intestinal BA receptors; **(C)** The mRNA expression of BA transporters; **(D)** Intestinal tight junction protein expression; **(E)** Serum BA profile; **(F,G)** The alteration of secondary BA species; **(H)** The correlation of altered secondary BAs and the genes regulating BA transport. Data are expressed as mean ± SD, *n* = 10 per group. ^*^ *p* < 0.05, ^**^ *p* < 0.01; ^***^ *p* < 0.001 between groups.

Physiologically, fecal BA reabsorption and excretion are in dynamic balance. BA undergoes continuous enterohepatic circulation, and 95% of BAs are re-absorbed at the ileum in each circle, thus the BA transport process determines the alteration of fecal BAs. IBAT is the chief mediator of intestinal BA absorption in charging transporting BA from the lumen to the enterocytes. The absorbed BAs in enterocytes then secret into the portal circulation *via* the basolateral BA transporters organic solute transporter subunit α (OST) α, OSTβ, multidrug-resistance protein (MRP) two and MRP3 (Figure 5A). BA receptors are reported to regulate the transporters, so we examined FXR and TGR5 expression in the intestine. Although the protein expression of FXR and TGR5 was decreased in NASH mice, HZRG intervention did not affect their expression, indicating the regulation of HZRG on BA receptors was limited (Figure 5B). The mRNA expression of IBAT and OSTβ was significantly increased in NASH mice compared to control mice, and HZRG intervention significantly decreased their expression. However, other transporters such as OSTα, MRP2, and MRP3 did not show statistical differences among groups (Figure 5C).

Suppression of BA transport indicates less BA absorption. Accumulation of toxic BAs may destroy the intestinal barrier. To verify the effect of HZRG on BA absorption, we first detected the tight junction proteins that are associated with intestinal permeability, and found that the expression of tight junction protein Occludin and Claudin was decreased in NASH mice, whereas HZRG intervention counteracted the decrease (Figure 5D). We further investigated the serum BA profiles of the mice to verify the regulation of HZRG on BA reabsorption. The total serum BA was significantly increased in NASH mice compared to control mice, whereas HZRG treatment reversed the increase of total serum BA level (Figure 5E). And the alteration of total serum BAs among 44 detected BA species was mostly attributed to the changes of secondary BA species. Most of the toxic DCA species showed an increase in NASH mice, and HZRG treatment significantly decrease the concentration of DCA, βDCA and GDCA (Figure 5F). Correspondingly, treatment of HZRG also decreased LCA, isoLCA and 6-ketoLCA levels of the NASH mice (Figure 5G). The altered secondary BAs were correlated with BA transport genes IBAT and OSTB (Figure 5H). Therefore, the decrease of serum secondary BAs upon HZRG treatment indicated lower reabsorption from the intestine, which was consistent with the alteration of fecal BA concentrations.

### Huazhi-Rougan Attenuates Hepatic Inflammation

**FIGURE 6:**
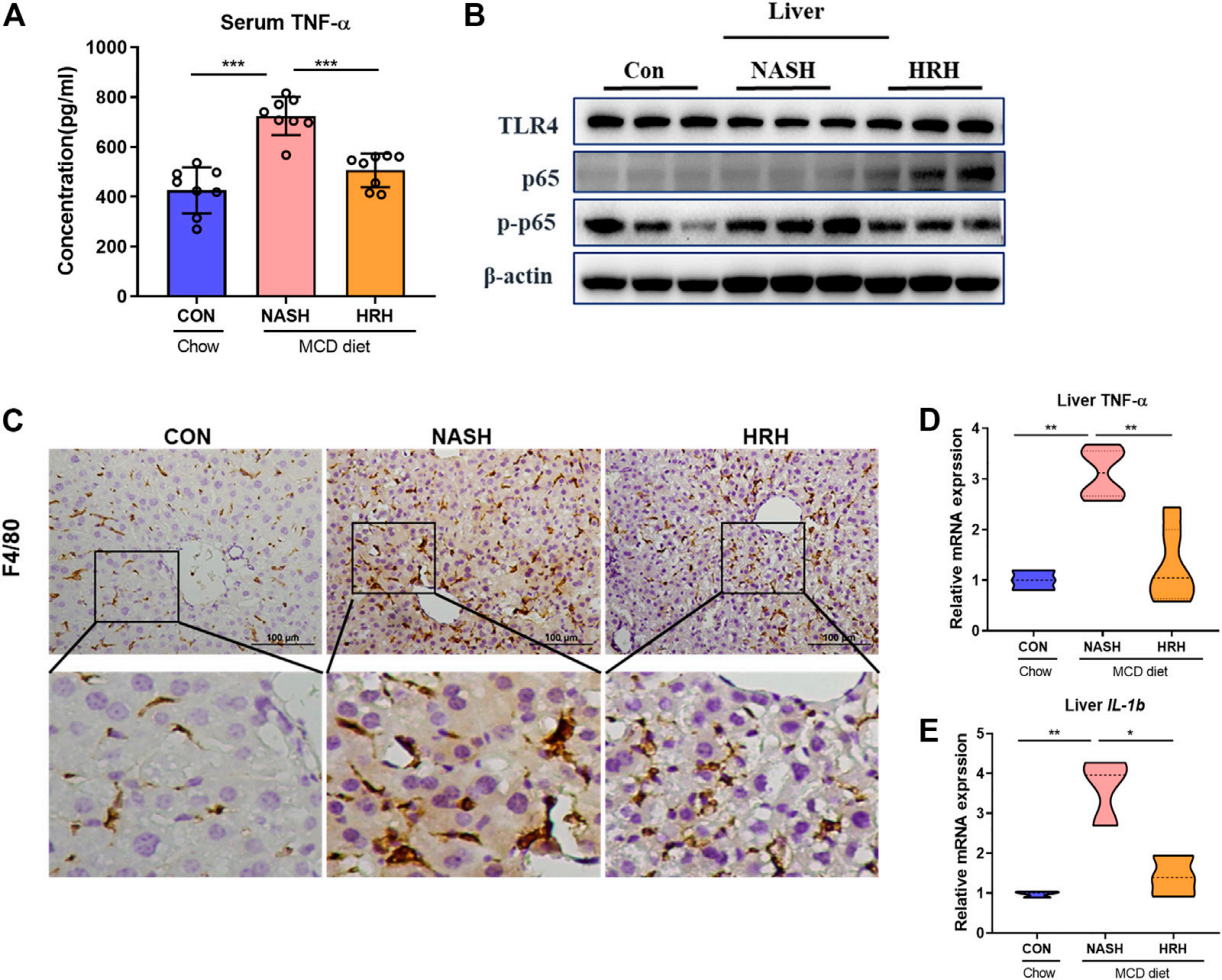
HZRG suppressed hepatic inflammation. **(A)** The serum concentration of TNF*α*; **(B)** The protein expression of liver TLR4, P-P65, P65. **(C)** IHC staining of liver macrophage biomarker F4/80; **(D,E)** The mRNA expression of liver *Tnfα* were and IL-1β. Data were shown as mean ± SD, ^*^ *p* < 0.05, ^**^ *p* < 0.01 between groups.

The suppression of toxic BA reabsorption along with the attenuation of the intestinal environment by HZRG is supposed to improve hepatic inflammation. HZRG significantly decreased the serum TNF-α level in NASH mice (Figure 6A). We also investigated a classic inflammatory pathway, and found that HZRG decreased the phosphorylation of nuclear factor-kappaB (NF-kB p65) (Figure 6B), indicating the inhibition of NF-kB activation. Consistently, the expression of macrophage marker F4/80 was increased in the liver of NASH mice, whereas HZRG treatment significantly reduced the expression of macrophage marker (Figure 6C). In addition, the mRNA of liver inflammatory cytokines TNF-α and interleukin-1 beta (IL-1β) also decreased upon HZRG treatment (Figures 6D,E). These results suggested that HZRG attenuated hepatic inflammation.

## Discussion

**FIGURE 7:**
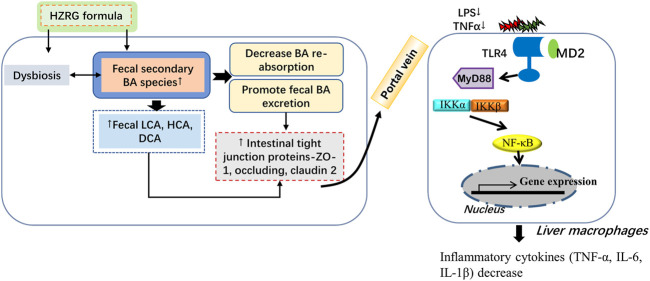
Graphic summary of the study.

TCM intervention is an important strategy for NASH treatment. The philosophy of TCM theories is different from modern medicine, which emphasizes an overview of the entire situation. The lack of recognized drugs is largely attributed to the complicated pathological mechanisms of NASH, and decades of works on exploring NASH drugs highlight that single target medicines are difficult to solve all problems (Friedman et al., 2018). The prescriptions with more than one medicinal that based on TCM theory have been widely used in Asian regions. In recent years, accumulating evidence has demonstrated the beneficial role of Chinese prescriptions in the treatment of NAFLD and NASH. HZRG prescription has been made as standard granules form, which is prescribed for NAFLD patients for decades. In the current study, we found that HZRG ameliorates the NASH phenotype induced by MCD diet feeding, the beneficial effects of HZRG are related to the modulation of gut dysbiosis and fecal BA excretion *via* inhibit IBAT expression (Figure 7).

HZRG is composed of 16 medicinal species that contain more than 100 ingredients. At this stage our analysis provides detailed qualitive analysis of the diversity of phytochemicals present. Any future clinical study should provide quantitative data on major constituents in the formula and perhaps information on their pharmacokinetics. According to TCM theory, the prescription is designed to clear Damp-Heat, while the best way to discard the pathogen is to promote their excretion. Analysis of HZRG revealed that the formula is rich in flavonoids, alkaloid and benzene, which were with low bioavailability, indicating the compounds might be abundantly detained in the intestine. Actually, the therapeutic effects of a series of TCM formulas are reported to be associated with the modulation of gut microbiota. Consistently, we found that HZRG modulated the gut dysbiosis, decreased the *Firmicutes*/*Bacteroidetes* (F/B) ratio in NASH mice. The F/B ratio increase is a feature of metabolic diseases, and regimens decreasing the ratio are reported to reverse the dysfunction (Spychala et al., 2018). The abundance of *Atopobiaceae* is reported to be associated with multidrug-resistant organism colonization in nursing home residents (Ducarmon et al., 2021). Enrichment of *Erysipelotrichaceae*, *Erysipelotrichaceae*, and *Streptococcaceae* was found in inflammatory bowel disease, obesity, NAFLD patients and animals, whereas decrease their abundant is associated with the alleviation of colitis (Lupp et al., 2007; Etxeberria et al., 2015; Monk et al., 2016; Sookoian et al., 2020). Consistently, we found that abundance of these pathogenic bacteria was all increased in NASH mice, where HZRG intervention significantly decreased their enrichment. HZRG specifically increased the enrichment of *Lactobacillaceae*, *Bifidobacteriaceae*, *Clostruduaceae*, *Chostridiales VadinBB60 group*, *Corynebacteriaceae*, *Solanales*, *Propionibacteriaceae*, *Micrococcaceae*, and *Satphylococcaceae*. The secondary BAs are formed from primary BAs, and numerous bacterial genera in the gut are involved in the BA transformation. *Bacteroides*, *Clostridium*, *Lactobacillus*, and *Bifidobacterium* are confirmed to carry out the deconjugation, oxidation and epimerization of BAs, and subsequently, *Bacteroides*, *Clostridium*, *Eubacterium*, *Lactobacillus* and *Escherichia* convert the unconjugated CDCA and CA into the secondary LCA and DCA (Ridlon et al., 2006). In our study, we noticed the increase of bacterial genera that contributes to secondary BA formation in HZRG treatment mice, which was consistent with the fecal BA profile.

Ileal BA transport is an efficient system, accounting for 95% intraluminal BA reabsorption at the terminal ileum. IBAT, also known as apical sodium-dependent bile acid transporter (ASBT), and SLC10A2, is mainly expressed in the apical membrane of ileal enterocytes. IBAT determines the size of the BA pool and regulates the homeostasis of lipid metabolism. By reducing gut-derived BAs entering the liver, IBAT inhibitors may potentially reduce the liver damage in cholestatic liver disease, and IBAT inhibitors, such as maralixibat and odevixibat, are in clinical programs for treating pediatric cholestatic liver diseases (Karpen et al., 2020). In diet-induced NAFLD mice, IBAT inhibitor IMB17-15 is reported to alter the intestinal BA composition and mediate intestinal FGF15/19 pathway, which contributes to the improvement of NAFLD phenotype in mice (Ge et al., 2019). Another IBAT inhibitor SC-435 is found to significantly reduce the liver fat of NAFLD mice, which is associated with liver FXR activation and lipid synthesis inhibition (Rao et al., 2016; Rao et al., 2020). Thus, IBAT inhibition presents as a new type of treatment strategy for NASH and related complications. In comparison to the currently under-testing FXR agonist obeticholic acid on NASH, specific inhibition of IBAT may reduce itching and other side effects (Li and Chiang, 2020).

The secondary BAs LCA and DCA are hydrophobic and unconjugated, and considered to be cytotoxic, which are related to intestinal barrier damage and liver cell injury. It is reported that the excretion of the LCA and DCA in feces is increased upon the application of IBAT inhibitor volixibat. Consistently, obese and overweight adults who orally take volixibat showed improved dyslipidemia accompanied by increased fecal BA excretion (Salic et al., 2019). Inhibition of IBAT also leads to increased BA delivery to the colon, which accelerates colonic transit and increases colonic secretion, thus regarded as a promising regimen for chronic constipation (Chedid et al., 2018). In the present study, we found that HZRG obviously suppressed the expression of IBAT in the intestine, and simultaneously reduced the expression of MRP2/3 and OSTα/β, the molecules that control BA transport back the portal vein.

BAs obtain potential cytotoxic effects on extra-hepatic tissues. The enterohepatic circulation functions to safely store and then promptly deliver BAs in high concentration to the intestinal lumen for digestion and absorption of lipids. The gut microbiota dysbiosis promoted BA homeostasis disbalance, characterized by the accumulation of LCA and DCA in feces (Guo et al., 2021). Dysregulated expression of the ASBT and OSTα/β may lead to BA accumulation and injury in liver epithelial cells. Higher levels of intestinal secondary BAs (DCA and LCA) are associated with the down-regulation of tight junction proteins, indicating that DCA and LCA impair gut barrier function (Pi et al., 2020). ASBT knockout mice showed more than 5-fold fecal BA excretion, reduced whole body BA pool size, and altered BA pool composition. Inactivation of OSTα in mice demonstrated the almost same extent of BA pool size as ASBT knockout mice (Dawson et al., 2003; Rao et al., 2008). Loss-of-function ASBT mutations in humans yield a classical primary BA malabsorption phenotype without ileal histological changes, suggesting IBAT inhibition potentiates protective effects on the intestinal environment (Oelkers et al., 1997).

The alteration of BAs may also regulate the intestinal immune response (Wang et al., 2021b). Interleukin -17 is a main pro-inflammatory cytokine involved in gut dysbiosis, and this role is highlighted by recent data indicating that the IL-17/IL-17R axis drives intestinal neutrophil migration, limits gut dysbiosis and attenuates LPS translocation to the circulation and tissue, resulting in protection to high-fat diet-induced mice (Perez et al., 2019). Actually, there is a previous study emphasizing the IL-17 role in this setting. Specifically, the association found between the amount of visceral fat and circulating levels of eotaxin on the one hand, and intima-media thickness on the other, could reinforce the hypothesis that IL-17, released by the visceral adipose tissue, induces eotaxin secretion *via* the smooth muscle cells present in the atheromatosus vessels of patients suffering from obesity-related NAFLD (Tarantino et al., 2014). Therefore, the role of BAs in maintaining the IL-17/IL-17R axis is also contribute to the protection effects of HZRG on NASH.

In conclusion, the current study demonstrates that HZRG treatment counteracts MCD-induced liver steatosis and inflammation, the beneficial effects of HZRG are associated with the modulation of gut dysbiosis and promotion of fecal BA excretion (Figure 7). Our findings expand the current knowledge of microbiota-BA interaction, and provide evidence that HZRG administration reduces pathogenic microbiota, enhances fecal BA excretion, and reduces secondary BA accumulation in the intestine.

## Data Availability

The datasets presented in this study can be found in online repositories. The names of the repository/repositories and accession number(s) can be found below: https://www.ncbi.nlm.nih.gov/bioproject/, PRJNA799458.

## Glossary

BA: bile acid
CYP7A1: cholesterol 7α-hydroxylase
CYP27A1: sterol-27-hydroxylase
CDCA: Chenodeoxycholic acid
CA: Cholic acid
CYP8B1: sterol 12α-hydroxylase
MCAs: muricholic acids
LCA: ithocholic acid
DCA: deoxycholic acid
HCA: hyocholicacid
HDCA: hyodeoxycholic acid
IBAT: ileal bile acid transporter
TCM: Traditional Chinese medicine
MCD: methionine-and choline-deficient
CMC-Na: carboxymethyl cellulose sodium
H&E: hematoxylineosin
ORO: Oil Red O
ALT: alanine aminotransferase
AST: aspartate aminotransferase
TNF-α: tumor necrosis factor-alpha
TG: triglyceride
TC: total cholesterol
RT-qPCR: Real-time quantitative PCR
PCoA: principal coordinates analysis
OSTβ: organic solute transporter subunit β
MRP2: Multidrug Resistanceassociated Protein 2
MRP3: Multidrug Resistanceassociated Protein 3
NF-kB: nuclear factor-kappaB
IL-1β: Interlukin-1 beta
F/B: Firmicutes/Bacteroidetes
ASBT: apical sodium-dependent bile acid transporter
SLC10A2: Solute Carrier Family 10 Member 2
