# Supplementary figures and images for: Medicinal Formula Huazhi-Rougan Attenuates Non-Alcoholic Steatohepatitis Through Enhancing Fecal Bile Acid Excretion in Mice
Source: Front Pharmacol. 2022 Jun 1;13:833414. doi: 10.3389/fphar.2022.833414 (PMC9198489; doi:10.3389/fphar.2022.833414)

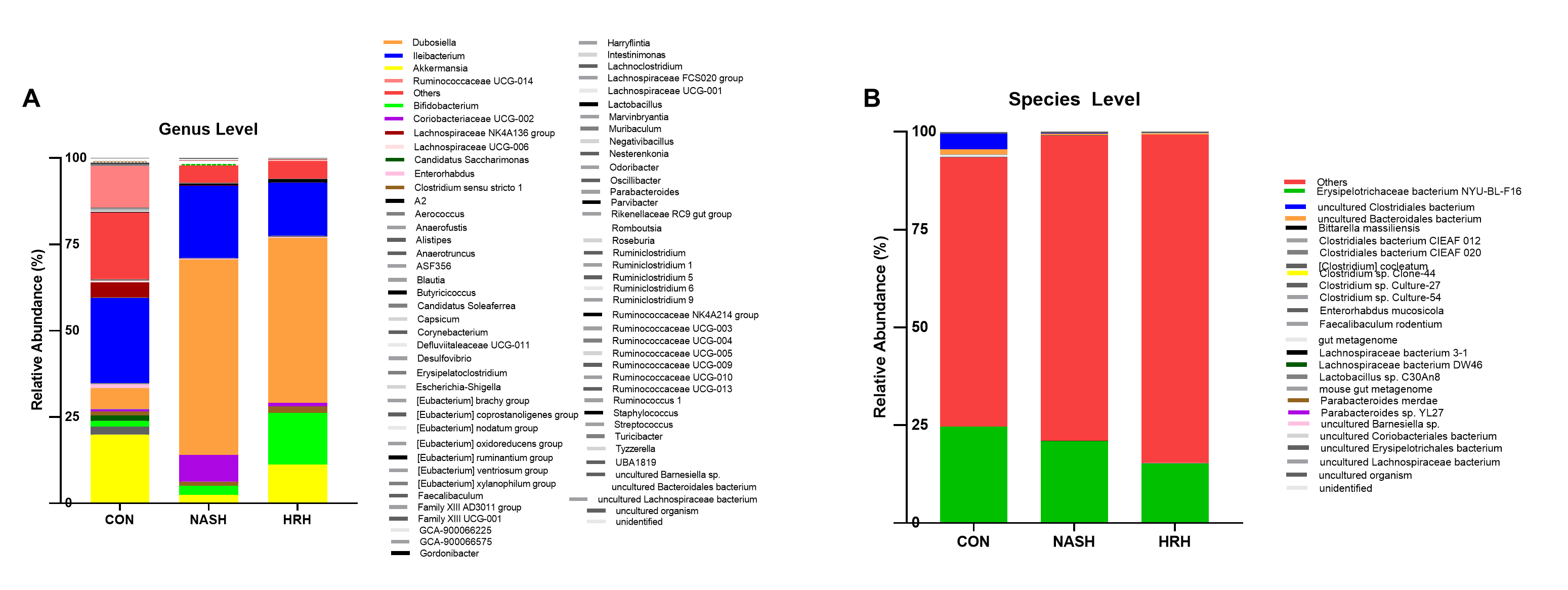

Supplement: Supplementary file 1 [file Image1.TIF]
